# Adaptive evolution of *Pseudomonas putida* in the presence of fluoride exposes novel functions of a benzoate transporter

**DOI:** 10.1128/jb.00479-25

**Published:** 2026-04-01

**Authors:** Lea Ets, Heili Ilves, Lisette Juhe, Tanel Ilmjärv, Òscar Puiggené, Pablo Iván Nikel, Maia Kivisaar

**Affiliations:** 1 Institute of Molecular and Cell Biology, University of Tartu 37546, Tartu, Estonia; 2 The Novo Nordisk Foundation Biotechnology Research Institute for the Green Transition, Technical University of Denmark 5205, Kongens Lyngby, Denmark; Dartmouth College Geisel School of Medicine, Hanover, New Hampshire, USA

**Keywords:** fluoride tolerance mechanisms, *Pseudomonas putida*, CrcB export protein, compensatory tolerance mechanisms, benzoate transporter BenE-I

## Abstract

Fluoride (F^−^), the anionic form of fluorine and the 13th most abundant element in Earth’s crust, is toxic to most organisms above relatively low threshold concentrations. Environmental bacteria often tolerate elevated fluoride levels, and one of the known resistance mechanisms involves CrcB-mediated efflux. In the environmental bacterium *Pseudomonas putida*, CrcB export is the primary defense against fluoride stress. However, spontaneous NaF-tolerant mutants emerge even without this channel, suggesting the existence of additional pathways. To uncover new mechanisms of fluoride tolerance, we performed a genome-wide screen of over 141,000 transposon mutants. We identified PP_3125, a Cro/cI-type transcriptional regulator, as essential for high fluoride tolerance in a Δ*crcB* background. Transcriptomic and proteomic analyses revealed PP_3125-regulated genes, including a gene encoding the benzoate transporter BenE-I, which contributes to fluoride tolerance. These findings demonstrate that bacterial transporters can acquire broader functions beyond their canonical roles and reveal previously unrecognized fluoride tolerance strategies in *P. putida*. Together, our results expand the understanding of microbial adaptation to toxic ions and provide new targets for engineering stress-resilient strains for environmental and industrial applications.

**IMPORTANCE:**

Our work identifies a new fluoride tolerance mechanism in *Pseudomonas putida* that functions independently of the well-characterized CrcB efflux system. We show that inactivation of transcriptional regulator PP_3125 activates a transporter with an unexpected moonlighting role in fluoride tolerance, highlighting how bacteria can repurpose existing functions to survive environmental stress. This discovery deepens our understanding of microbial stress responses and suggests strategies to engineer robust microbial strains capable of thriving in fluoride-contaminated settings. Such strains could be valuable for bioremediation, sustainable bioprocessing, and other biotechnological applications where fluoride exposure limits microbial performance.

## INTRODUCTION

Microorganisms frequently encounter environmental stressors, including heavy metals, organic solvents, and toxic anions, which necessitate various adaptive mechanisms for survival and growth. One key aspect of microbial resilience is chemical tolerance—the capacity of cells to detect, mitigate, and adapt to toxic compounds. Bacteria have evolved diverse strategies to cope with such stresses, including the activation of efflux transporters, modification of membrane permeability, enzymatic detoxification, and reprogramming of cellular metabolism (1, 2). Understanding these mechanisms has significant implications for biotechnology, remediation of environmental pollutants, and synthetic biology, where engineered microbes are often exposed to non-natural or stressful substrates.

Fluoride, one of the most common environmental halogens (3), is a pervasive toxicant to microbial life. At sublethal concentrations, fluoride disrupts metabolic processes by inhibiting key enzymes, such as enolase and pyrophosphatases, often by forming complexes with essential metal cofactors (4, 5). To counteract this, many microorganisms possess fluoride-specific ion channels, the best known of which are the CrcB channel and CLCF fluoride exporters. CrcB functions as a passive ion channel and is considered a primary defense mechanism against fluoride toxicity in many bacteria, including *Escherichia coli*, *Bacillus subtilis*, and *Pseudomonas* species (5–7). CLCF is an F^−^/H^+^ antiporter and is also found in many bacteria, including *Enterococcus casseliflavus* and *Streptococcus mutans* (8–12). Other, less-studied fluoride tolerance mechanisms include mutated enolase enzymes in some fluoride-tolerant *S. mutans* strains (13) and *rarA, lapA,* and *lapD* in *Pseudomonas putida* (14).

*P. putida* is a gram-negative environmental bacterium renowned for its metabolic versatility, solvent resistance, and capacity to degrade various xenobiotic compounds (15). Its physiological robustness has led to its widespread adoption as a model organism for environmental and industrial biotechnology. *P. putida* KT2440 is a well-characterized, genetically tractable strain chassis for metabolic engineering (16). One emerging area of interest is the use of *P. putida* in biofluorination—the biosynthesis of organofluoride compounds, which are otherwise chemically challenging to produce (7, 17, 18). This process often involves exposure to sodium fluoride (NaF) as a substrate or intermediate, underscoring the importance of engineering fluoride-tolerant microbial platforms (5, 7, 19).

Chemical tolerance in bacteria is frequently governed by transcriptional regulators that orchestrate the cellular stress response. Negative regulators can suppress the expression of tolerance-conferring genes, and their inactivation often leads to increased tolerance. One such regulator is PsrA. This transcriptional repressor regulates fatty acid metabolism. In its absence, the growth rate and polyhydroxyalkanoate (PHA) production are reduced, indicating its role in negatively regulating genes related to fatty acid metabolism (20). Additionally, multidrug and metabolite efflux transporters are known to contribute to the export of toxic compounds, including ions and solvents (2, 21). Such systems may play a role in fluoride tolerance, although their specific involvement remains understudied. As of yet, CrcB-mediated export is known to be the primary mechanism of fluoride tolerance in *P. putida*.

In this study, we report that the evolution of a Δ*crcB* derivative of *P. putida*, lacking the fluoride efflux pump, can give rise to spontaneous NaF-tolerant mutants, suggesting the existence of secondary or compensatory tolerance mechanisms. Through a combination of spontaneous NaF-tolerant mutant selection, transposon mutagenesis, targeted gene deletions, proteomic profiling, and transcriptomic analysis, we identified a previously uncharacterized transcriptional regulator, PP_3125, as a key modulator of fluoride sensitivity. We further demonstrated that inactivation of *PP_3125* derepresses five genes, including the gene encoding the benzoate transporter BenE-I, which contributed to elevated fluoride tolerance.

## RESULTS

### Genomic changes in spontaneous NaF^+^ mutants in the **Δ***crcB* strain

The ability of *P. putida* to tolerate high fluoride concentrations relies on the activity of the CrcB export protein as the main mechanism in this organism. On LB agar plates, wild-type KT2440 cells can tolerate fluoride concentrations even higher than 30 mM (Fig. 7), whereas the strain lacking the CrcB channel (Δ*crcB* strain) shows growth restrictions already at 0.25 mM of NaF (14). However, we observed the appearance of single colonies when the Δ*crcB* strain was incubated on LB plates containing 5 mM NaF. The spontaneous NaF-tolerant mutant frequency of the Δ*crcB* strain was estimated as specified in Materials and Methods. We observed that the median number of NaF-tolerant mutants that emerged at 5 mM NaF was 5 CFUmL^−1^. Four isolated mutants designated *crcB1*, *crcB4*, *crcB12*, and *crcB18* were sequenced to investigate the mechanisms underlying fluoride tolerance.

Comparative genomic analysis revealed that all four Na-tolerant Δ*crcB* mutants carried large deletions in their genomes, ranging from 18 kb to 340 kb (corresponding to 18–345 genes), as illustrated in Fig. S1. While the deleted genomic regions overlapped, their start and end points varied. The specific deleted areas were as follows: *crcB1* [*PP_2985*]-*PP_5598; crcB4* [*PP_2985*]-[*PP_3288*]; *crcB12* [*PP_3119*]-[*PP_3133*]; and *crcB18* [*PP_3059*]-[*PP_3221*], where genes with square brackets are partially deleted. It should be noted that in *P. putida*, locus tag numbering does not always correspond to chromosomal order, as newly identified genes are assigned the next available numbers during annotation. Consequently, *PP_5598* is not located at the end of the genome but lies within a central genomic region. The list of all the deleted genes of the shortest deletion can be found in Table S1.

Given the large deletions, which made it challenging to pinpoint the exact genes responsible for increased fluoride tolerance, we performed transposon mutagenesis using the mini-Tn*5* system to identify specific genes affecting fluoride tolerance in the Δ*crcB* strain.

### Identification of genes affecting fluoride tolerance in the *P. putida ΔcrcB* strain

**Fig 1:**
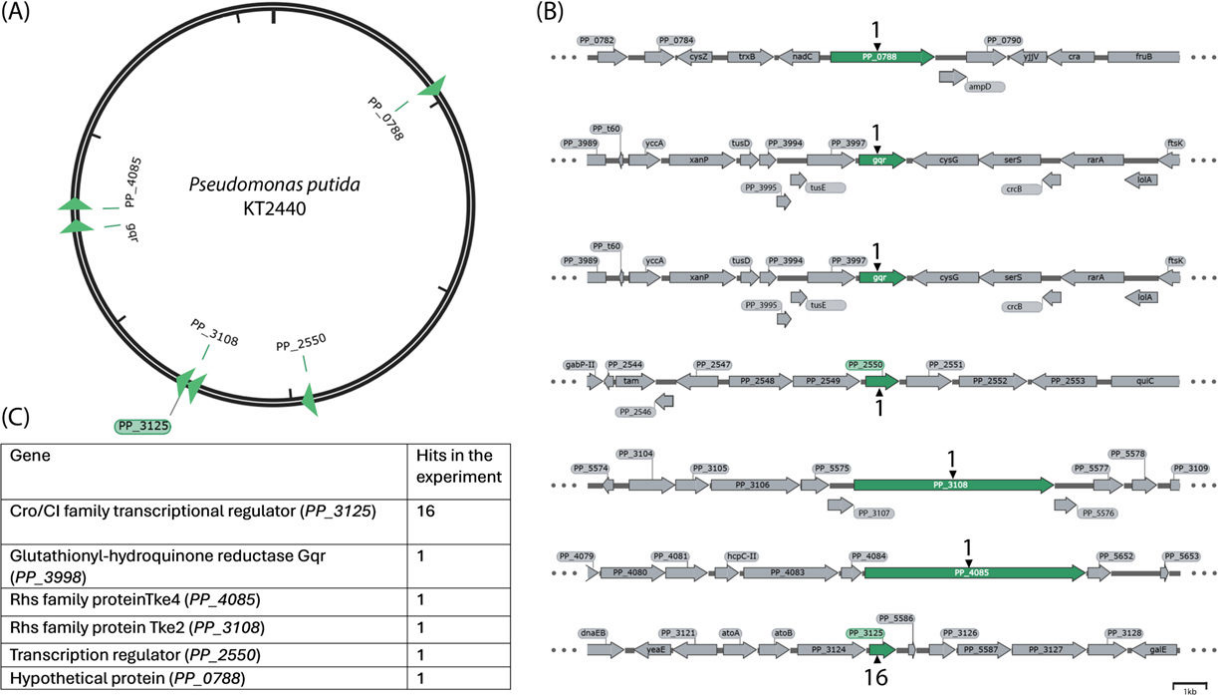
(**a**) Physical map of the genome of *P. putida* KT2440 illustrating the insertion sites of the transposon. The gene with the most hits, PP_3125, is marked with a green background. (**b**) Genetic context of transposon insertions and the count of transposon mutants. Genes containing transposon insertions are marked in green, the number of insertions is indicated with black arrowheads, and gray-marked genes represent the genetic context surrounding the genes with transposon insertions. (**c**) Isolated transposon mutants with gene descriptions and hits per gene.

To identify the genes affecting fluoride tolerance in *P. putida*, transposon mutagenesis was performed with the Δ*crcB* strain. Approximately 141,000 transposon mutants from three independent transposon mutagenesis experiments were obtained, and the fluoride tolerance of the mutants was analyzed on plates containing 5 mM NaF. Out of 141,000 mutants, 48 had developed higher fluoride tolerance. The chromosomal location of mini-Tn*5* was identified in 24 mutants. Mutations were located all over the genome, as shown in Fig. 1a, which indicates that there was no significant positional bias in the assay. As seen in Fig. 1b and c, from those 24 mutants analyzed, 16 contained the transposon insertions into the *PP_3125* gene. We focused on the *PP_3125* gene for further analysis, as all the other insertions targeted the other genes only once. It is worth noting that the *PP_3125* gene was always included in the DNA region that was deleted in the spontaneous NaF-tolerant Δ*crcB* mutants *crcB1*, *crcB4*, *crcB12*, and *crcB18*, as shown in Fig. S1.

Our results indicated that inactivation of *PP_3125* encoding Cro/CI type transcriptional regulator increases the fluoride tolerance of the Δ*crcB* strain.

### Involvement of the *PP_3125* gene in NaF tolerance of the Δ*crcB* strain

To confirm the effect of PP_3125 in fluoride tolerance, the following strains were constructed: Δ*3125—P. putida* KT2440 strain where *PP_3125* gene is deleted; Δ*crcB*Δ*3125—P. putida* KT2440 double mutant where both the genes *crcB* and *PP_3125* were deleted; and Δ*crcB*Δ*3125*-tac*3125*—double deletion strain with the gene cassette containing the *PP_3125* gene under the control of P*_tac_* promoter in the chromosome at the Tn*7* insertion site. The growth experiments in liquid media and on LB agar plates with different NaF concentrations were carried out. To analyze the growth of constructed strains on solid media, 10-fold serial dilutions of overnight cultures of *P. putida* strains were spotted on LB agar plates with NaF concentrations ranging from 0 to 5 mM NaF and analyzed after 20 h of growth. The liquid media growth experiments were carried out for 24 h under varying NaF concentrations (0, 0.5, and 2.5 mM NaF).

**Fig 2:**
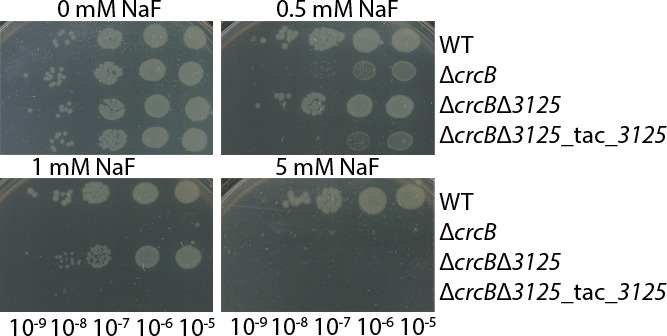
Dilution spot assay of *P. putida* WT, Δ*crcB*, Δ*3125*, Δ*crcB*Δ*3125,* and Δ*crcB*Δ*3125*_tac_*3125* strains on LB plates supplemented with 0–5 mM NaF. Growth on different fluoride concentrations was examined by spotting 10-fold serial dilutions of overnight cultures of the strains onto plates with different NaF concentrations. The plates were incubated at 30°C for 20 h.

**Fig 3:**
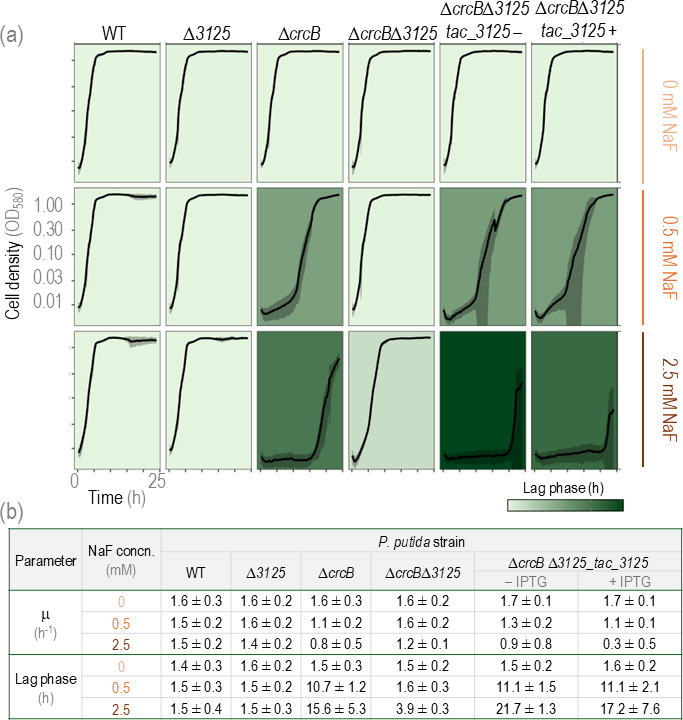
Growth of *P. putida* WT, Δ*crcB*, Δ*3125*, Δ*crcB*Δ*3125,* and Δ*crcB*Δ*3125_*tac_*3125* strains growing on different NaF concentrations. (**a**) Growth curves of *P. putida* WT, Δ*crcB*, Δ*3125*, Δ*crcB*Δ*3125,* and Δ*crcB*Δ*3125*_tac_*3125* strains growing on different NaF concentrations; 0.01 mM IPTG was used to induce the *tac* promoter. Averages of three different biological replicas with three different technical parallels are presented with standard deviation. The background colors show the length of the lag phase; the darker the background, the longer the lag phase. (**b**) Maximum growth rate (μ) and lag phase with standard deviation of *P. putida* WT, Δ*crcB*, Δ*3125*, Δ*crcB*Δ*3125,* and Δ*crcB*Δ*3125_*tac_*3125* strains growing on different NaF concentrations.

As shown in Fig. 2 and 3, in the absence of NaF, all strains exhibited similar growth patterns. However, in the presence of 0.5 mM NaF, the Δ*crcB* strain showed reduced growth on LB agar plates, whereas the Δ*crcB*Δ*3125* strain did not show any growth restrictions (Fig. 2). Also, in liquid media, at 0.5 mM NaF, the Δ*crcB* strain showed a longer *lag* phase than Δ*crcB*Δ*3125* (10.7 h vs. 1.6 h, adjusted *P*-value = 0.0293). With the higher fluoride concentrations, the differences were even more pronounced. The difference in the maximum growth rate was not significant, which shows that exposure of these strains to fluoride affects the length of the *lag* phase but not the growth rate.

Due to leaky expression from the P*_tac_* promoter, the Δ*crcB*Δ*3125*-tac*3125* strain showed similar growth to the Δ*crcB* strain in NaF-containing media, even without IPTG induction (Fig. 2 and 3). The high variability in growth curves of fluoride-sensitive strains in liquid media at the end of the experiment could be attributed to the emergence of spontaneous mutants capable of tolerating elevated fluoride concentrations.

### Spontaneous NaF-tolerant Δ*crcB* mutants have similar growth characteristics to the Δ*crcB*Δ*3125* strain

Since *PP_3125* is located within the deleted regions of the spontaneously occurring NaF-tolerant Δ*crcB* mutants, we investigated whether the deletion of *PP_3125* alone had a similar effect on NaF tolerance as that observed in the spontaneous mutants.

**Fig 4:**
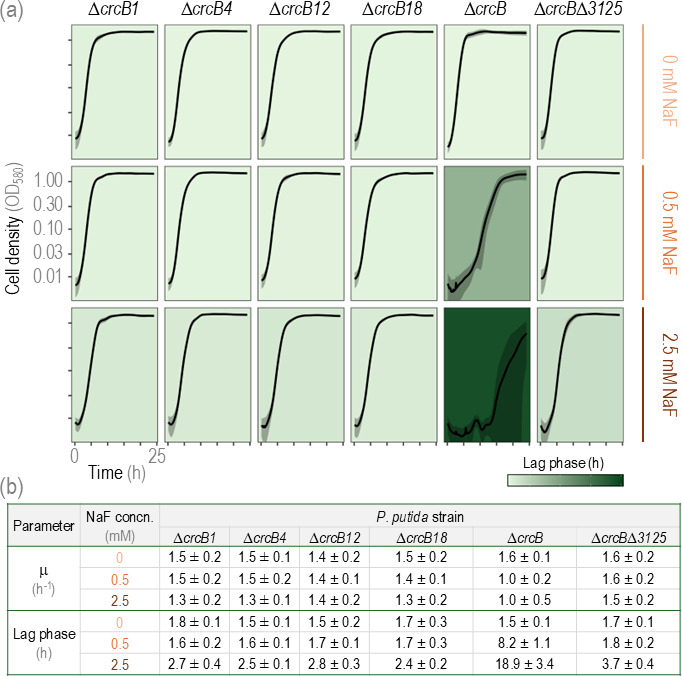
Growth of *P. putida* Δ*crcB*, Δ*crcB*Δ*3125*, and spontaneous NaF+ Δ*crcB* mutants crcB1, 12, 18, and 4 growing on different NaF concentrations. (**a**) Growth curves of *P. putida* Δ*crcB*, Δ*crcB*Δ*3125*, and spontaneous NaF+ ΔcrcB mutants crcB1, 12, 18, and 4 growing on different NaF concentrations. Averages of three different biological replicas with three different technical parallels are presented with standard deviation. The background colors show the length of the *lag* phase*;* the darker the background, the longer the lag phase. (**b**) Maximum growth rate (μ) and lag phase with standard deviation of *P. putida* Δ*crcB*, Δ*crcB*Δ*3125*, and spontaneous NaF+ ΔcrcB mutants crcB1, 12, 18, and 4 growing on different NaF concentrations.

As shown in Fig. 4, the spontaneous mutants exhibited growth comparable to the wild-type (WT) strain in the absence of NaF. However, when NaF was added, their growth patterns closely resembled the growth of the Δ*crcB*Δ*3125* strain. When the *lag* phase of the Δ*crcB* strain with 2.5 mM NaF was 18.9 h long, the *lag* phase of the spontaneous mutants and the Δ*crcB*Δ*3125* strain was seven times shorter, but the differences were not significant. This might be due to a very high variability in the growth pattern of the Δ*crcB*. However, the results still indicate that the NaF tolerance observed in the spontaneous mutants is most probably due to the absence of *PP_3125*.

### Identification of genes regulated by PP_3125

A proteomic analysis was conducted to identify the genes regulated by PP_3125 that might contribute to fluoride tolerance. All strains were grown under different NaF concentrations, resulting in similar growth effects, specifically a doubling of the *lag* phase compared to the growth without NaF. For the Δ*crcB* strain, 0.2 mM NaF; for the Δ*crcB*Δ*3125* strain, 3.5 mM NaF; for the Δ*3125* strain, 30 mM NaF; and for the WT strain, 33 mM NaF concentrations were used.

**Fig 5:**
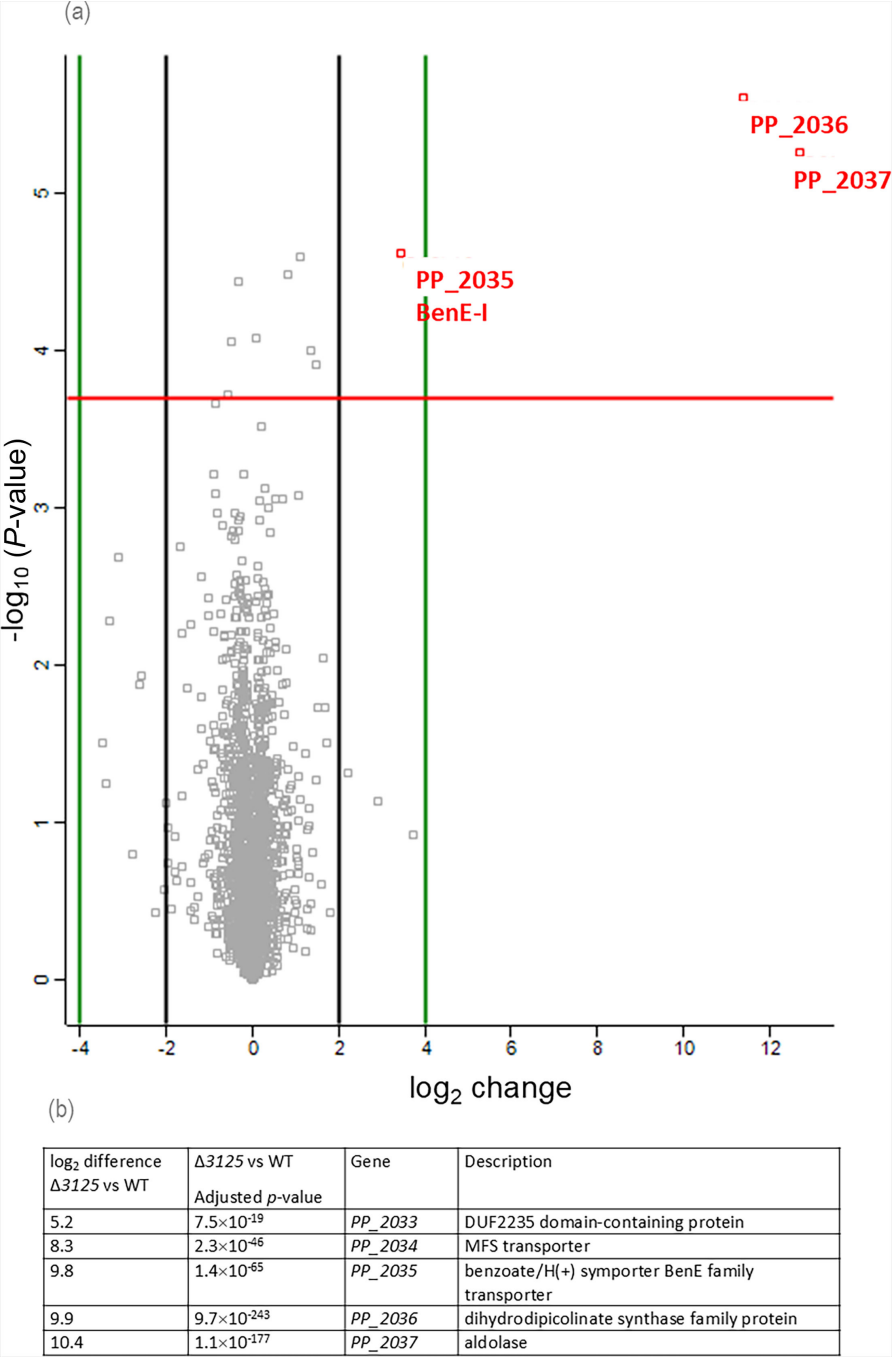
Overview of *P. putida* WT and Δ*3125* strains' full proteome and transcriptome comparison in the presence of NaF. (**a**) Proteomics analysis. In a volcano plot, every dot represents a protein. The horizontal line indicates the statistical significance threshold after Benjamin–Hochberg multiple testing correction (false discovery rate [FDR] = 0.05). The vertical lines indicate a 2-fold difference between the compared proteomes. Statistically significant at least 2-fold increase in protein level is presented as red dots with the gene name. (**b**) Transcriptome analysis of *P. putida* genes in the absence of PP_3125.

As shown in Fig. 5a, in the presence of NaF, only three proteins exhibited significantly differential expression when comparing the *PP_3125* deletion strain to the WT strain in the presence of fluoride. The overproduced proteins were PP_2036 (4-hydroxy-tetrahydrodipicolinate synthase), PP_2037 (aldolase), and BenE-I (PP_2035, benzoate transport protein). As these same protein genes were also overexpressed in the Δ*crcB*Δ*3125* strain compared to both the WT (Fig. S2) and the *ΔcrcB* strains in the presence of NaF (Fig. S3), this suggests that NaF may regulate *PP_3125* expression.

To further explore which other genes could also be regulated by PP_3125, the transcriptome of the WT, Δ*crcB*, Δ*3125,* and Δ*crcB*Δ*3125* strains was prepared under the same conditions as those performed in proteome studies.

Transcriptome analysis revealed the distinct effects of fluoride, *crcB* deletion, and *PP_3125* deletion (Fig. 5b). When PP_3125 proficient strains were compared to the strain lacking the regulator PP_3125 in the presence of fluoride in the growth medium, we were able to confirm only the differential expression of five genes. Other differently expressed genes resulted from the fluoride stress, as these genes were also differently expressed in the *crcB* deletion strain (Fig. S1). Five genes overexpressed in the absence of *PP_3125* are located in the same genomic region, adjacent to each other. This indicated that these genes could be co-regulated. Importantly, among the five identified genes, *PP_2036*, *PP_2037*, and *benE-I* (*PP_2035*) were also overexpressed in the proteomic analysis. The other two genes, *PP_2034* (a hypothetical transporter) and *PP_2033* (of unknown function), were clustered in the same region. The results of the transcriptome analysis can be found in Table S2.

### PP_3125-regulated genes affecting fluoride tolerance

To determine which of the five genes identified in the transcriptomic study could affect fluoride tolerance, a deletion strain lacking all five genes (PP_2033-PP_2037) was first constructed. As two out of five genes in the operon were transporters (*PP_2034* and *benE-I*), we suspected that one of the transporters could also transport fluoride in addition to its primary substrate. We first constructed plasmid-based overexpression constructs of these two genes, where these are under the control of a P*_tac_* promoter.

**Fig 6:**
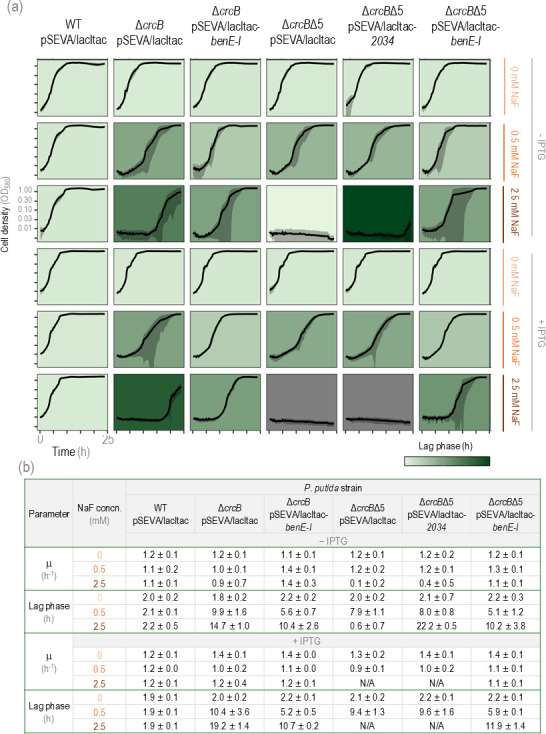
Growth of *P. putida* WT, Δ*crcB,* and Δ*crcB*Δ*5* with empty pSEVA/lacItac plasmid or with *PP_2034* or *benE-I* in a plasmid under the control of P*_tac_* growing on different NaF concentrations and with or without 0.1 mM IPTG. (**a**) Growth curve of *P. putida* WT, Δ*crcB,* and Δ*crcB*Δ*5* with empty pSEVA/lacItac plasmid or with *PP_2034* or *benE-I* in a plasmid under the control of P*_tac_* growing on different NaF concentrations and with or without 0.1 mM IPTG. Averages of three different biological replicas with three different technical parallels are presented with standard deviation. The background colors show the length of the *lag* phase; the darker the background, the longer the *lag* phase. In total, 0.1 mM of IPTG is added as an inducer. (**b**) Maximum growth rate (μ) and lag phase with standard deviation of *P. putida* WT, Δ*crcB,* and Δ*crcB*Δ*5* with empty pSEVA/lacItac plasmid or with *PP_2034* or *benE-I* in a plasmid under the control of P*_tac_* growing on different NaF concentrations and with or without 0.1 mM IPTG.

Due to leaky expression from the P*_tac_* promoter, the effect of the transporters was observed even in the absence of IPTG. As shown in Fig. 6, the Δ*crcB*Δ5 strain lacking all five genes identified in the transcriptomics study (*PP_2033-PP_2037*) could not grow in the presence of 2.5 mM NaF, even after 24 h. Introducing the *PP_2034* transporter into the Δ*crcB*Δ5 strain resulted in minimal growth toward the end of the experiment; however, this growth was minimal. In contrast, introducing the BenE-I transporter significantly improved the fluoride tolerance of the Δ*crcB*Δ5 strain, allowing growth at 2.5 mM NaF. Although expressing *benE-I* did not restore the fluoride tolerance to the wild-type levels, the tolerance was notably higher than that of the Δ*crcB* strain. Additionally, when the BenE-I transporter-encoding gene under the P*_tac_* promoter control was introduced into the Δ*crcB* strain, a shorter lag phase was observed, compared to the Δ*crcB* strain without the BenE-I transporter.

The addition of IPTG into the growth medium of bacteria further amplified the impact of the five-gene deletion, leading to even more pronounced growth inhibition by NaF. However, we observed that IPTG itself affected bacterial growth, as the Δ*crcB* strain with an empty plasmid exhibited a longer lag phase in the presence of IPTG compared to growth without IPTG supplementation (14.7 h without IPTG vs. 19.2 h with IPTG; however, no statistically significant difference was observed). The deletion of *PP_2037*, the aldolase gene, reduced the fluoride tolerance, but the overexpression did not restore the tolerance (Fig. S4). As the aldolase gene belongs to the same operon as *benE-I,* its deletion could negatively affect the expression of *benE-I*, which is located downstream of the aldolase gene. Therefore, we did not observe a direct effect of aldolase on fluoride tolerance.

**Fig 7:**
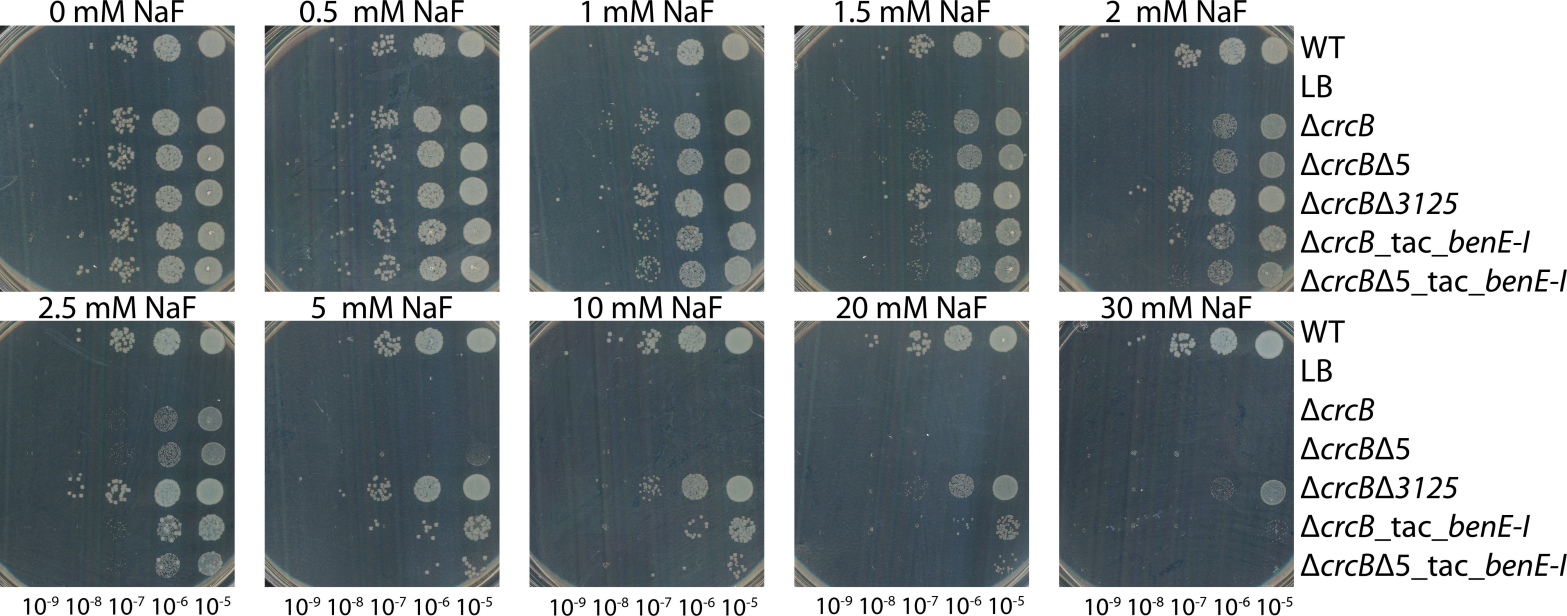
Dilution spot assay of *P. putida* WT, Δ*crcB*Δ5, Δ*crcB*Δ*3125,* Δ*crcB_*tac_*benE-I,* and Δ*crcB*Δ5_tac_*benEI* on LB-Km plates supplemented with 0–30 mM NaF and 0.1 mM IPTG as P_tac_-promoter inducer. Growth on different fluoride concentrations was examined by spotting 10-fold serial dilutions of overnight cultures of the strains onto plates with different NaF concentrations. The plates were incubated at 30°C for 20 h.

To compare the growth of *P. putida* KT2440 wild-type strain, Δ*crcB* strain, and its derivatives when either the *PP_3125* regulator gene or five genes identified in a transcriptomics study (*PP_2033-PP_2037*) were removed additionally, and the strains with the plasmid containing a *benE-I* overexpression construct, a growth assay on LB agar plates was carried out. All the compared strains contained either an empty pSEVA/lacItac plasmid or a pSEVA/lacItac-*benE-I* plasmid. The results shown in Fig. 7 demonstrated that all strains grew equally well on LB media without NaF. Strains lacking the CrcB channel did not grow on 5 mM NaF, whereas the WT strain tolerated over 30 mM NaF. Removing transcription regulator PP_3125 increased fluoride tolerance of the Δ*crcB* strain, as the strain missing both CrcB and PP_3125 was able to grow on 5 mM NaF, as well as the wild-type strain. As indicated by the growth in liquid media (Fig. 6), PP_3125 could be the negative regulator of BenE-I. When this regulator is absent, the expression of the *benE-I* transporter is higher, and bacteria can tolerate higher concentrations of NaF. Moreover, we observed that when the BenE-I transporter was artificially overproduced under the control of the *P_tac_* promoter in the Δ*crcB* strain or in the Δ*crcB*Δ5 strain, the NaF tolerance increased further, allowing bacterial growth even at 20 mM NaF (Fig. 7). In Fig. 6, for the Δ*crcB*Δ*5* strain carrying an empty plasmid on 2.5 mM NaF without IPTG, there was a slight variation in the optical density measurements at the beginning of the experiment, which was interpreted as the start of growth, and thus, the lag phase is not shown to be short, although there is no real growth.

### Effect of *benE-I* overexpression on intracellular fluoride concentration

To analyze the effect of the BenE-I on fluoride tolerance, the fluoride-sensitive riboswitch (FRS) biosensor (7) was used to estimate the amount of intracellular fluoride in the WT strain, in the Δ*crcB* strain, and in strains where *benE-I* has been deleted (Δ*crcB*Δ*benE-I*) and reintroduced under the control of the *P_tac_* promoter (Δ*crcB*Δ*benE-I*_tac_*benE-I*)

**Fig 8:**
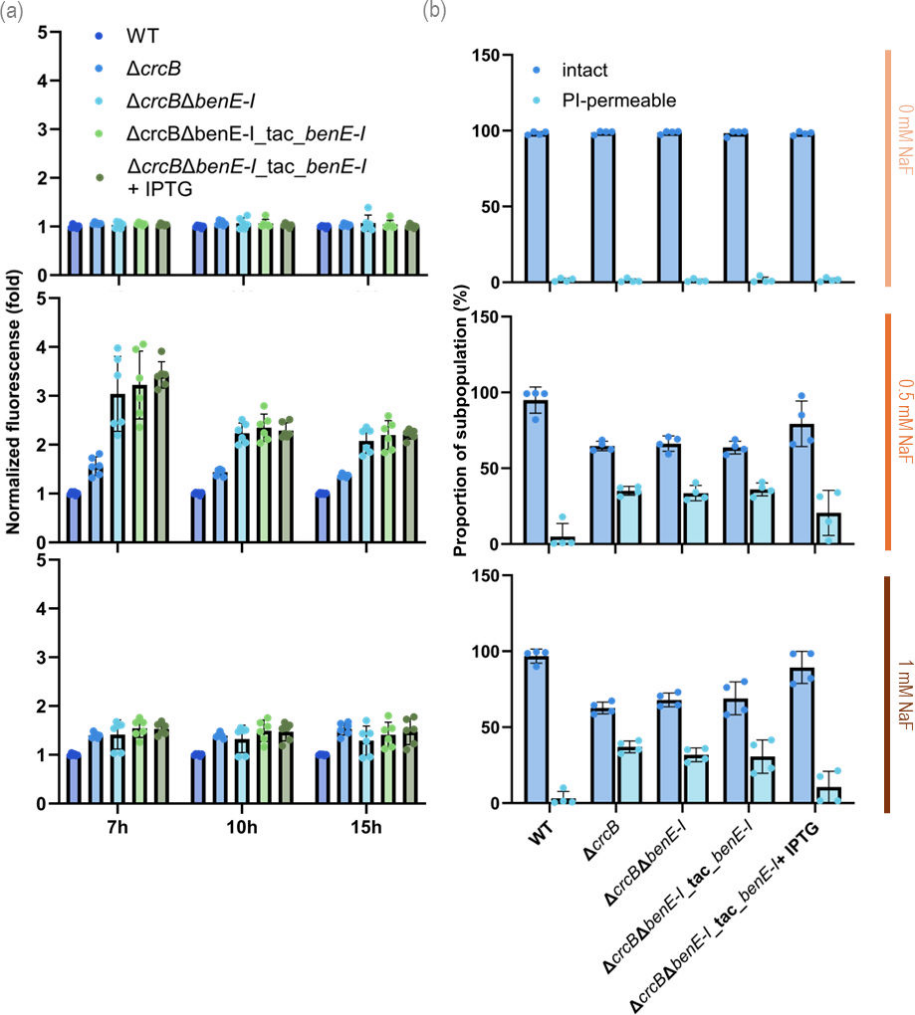
(**a**) Intracellular F^−^ concentration estimated with a biosensor based on the fluoride-sensitive riboswitch (FRS), which couples the presence of F^−^ with a fluorescent (msfGFP) output. Normalized msfGFP fluorescence (indicated as the fold change) of the individual *P. putida* strains carrying the FRS biosensor compared to the WT strain incubated in the presence of different NaF concentrations (0, 0.25, 1, and 15 mM). Measurements were done after 7, 10, or 15 h of incubation. In all cases, error bars represent standard deviations of average values calculated from at least three independent biological replicates. (**b**) Flow cytometry analysis of strains carrying the fluoride biosensor stained with SYTO9 and propidium iodide (PI) at different NaF concentrations. Relative proportions of the subpopulations of intact and PI-permeable cells (means with standard deviations) from two biological replicas are presented. The measurement was done after 15 h of fluoride incubation.

As presented in Fig. 8a, the deletion of *benE-I* elevates the intracellular fluoride concentration, but the overexpression of this gene does not lower it. The intracellular fluoride is probably higher due to more permeable cell membranes, as seen in Fig. 8b. The effect on membrane permeability is only visible in the presence of NaF. The population with a permeable membrane is larger in the *benE-I* deletion strains and slightly smaller in the *benE-I* overexpression strains, but the difference is not statistically significant.

Overall, these results demonstrate that PP_3125 functions as a negative regulator of a gene influencing fluoride tolerance in *P. putida*. BenE-I, a benzoate transporter regulated by PP_3125, enhances fluoride tolerance when overexpressed. Naturally occurring NaF-tolerant Δ*crcB* mutants appeared to gain their tolerance through deletion of a genomic region containing *PP_3125*, as this gene was absent in all sequenced spontaneous mutants, and the fluoride tolerance levels of spontaneous mutants and the Δ*crcB*Δ*3125* deletion strain were comparable. The benzoate transporter BenE-I has a role in fluoride tolerance, as its overexpression enables strains with lower fluoride tolerance to tolerate higher fluoride concentrations but does not decrease intracellular fluoride concentrations.

## DISCUSSION

The findings of this study significantly advance our understanding of fluoride tolerance mechanisms in *P. putida*. Previous studies have highlighted the role of the CrcB export protein (also known as Fluc) in exporting intracellular fluoride ions (7). This work uncovers an additional, CrcB-independent pathway involving the transcriptional regulator PP_3125. Deletion of *PP_3125* in the Δ*crcB* background led to a marked increase in fluoride tolerance (Fig. 3), mirroring the phenotype observed in spontaneous fluoride-tolerant mutants with large genomic deletions encompassing this gene (Fig. 4).

There are two distinct classes of fluoride export proteins: ClCF and CrcB (Fluc) channels (5, 6, 22). ClCF transporters function as proton-fluoride antiporters, facilitating active extrusion of fluoride from the cell (22). In contrast, CrcB export proteins function as passive fluoride-specific ion channels that require Na^+^ ions for transport activity (4, 5, 23–26). While ClCF transporters can transport both chloride and fluoride ions or only fluoride, CrcB channels are highly selective for fluoride (6, 12). These mechanistic distinctions underscore the importance of identifying alternative fluoride tolerance strategies when CrcB is deleted or nonfunctional.

Using transposon mutagenesis, we identified *PP_3125* as the most frequently disrupted gene in fluoride-tolerant Δ*crcB* mutants, indicating its central role as a negative regulator of the fluoride stress response. Proteomic and transcriptomic analyses further revealed that PP_3125 negatively regulates *PP_2033* and an adjacent operon of four genes (*PP_2034–PP_2037*), including transporters and metabolic enzymes. Among these genes, *PP_2035*, encoding for BenE-I transporter, a member of the benzoate/H^+^ symporter family, emerged as a key factor in promoting fluoride tolerance when overexpressed. An increase in fluoride tolerance in Δ*crcB* strains occurred through the overexpression of this gene, both in the solid medium (Fig. 7) and in the liquid medium (Fig. 6) when we observed that the lag phase of the BenE-I complementation strain was shorter than that of the deletion strain (Fig. 6). The lag phase is the growth parameter most strongly affected by the fluoride stress (27). Although deletion of *PP_2037,* aldolase, did lengthen the lag phase, the overexpression of the gene did not have any effect on the growth. As the aldolase gene is located upstream of the benzoate transporter gene, it is possible that this deletion affected the expression of *benE-I* (Fig. S4).

*P. putida* is known for its capacity to metabolize a wide range of aromatic compounds, including benzoate. Key transporters involved in benzoate transport are BenK, BenE, and BenF. BenK and BenE function as aromatic acid-H^+^ symporters, while BenF operates as a benzoate-specific porin and efflux pump (28, 29). The functional redundancy and substrate specificity of these transporters reflect the organism’s metabolic adaptability. The functioning of BenE1-I as a benzoate transporter has been reported in previous studies (28, 29). Is it possible that BenE-I could be involved in addition to benzoate, also in fluoride transport? The experiments performed by us did not support this possibility. We used the GFP-based fluoride biosensor to monitor intracellular fluoride concentration in *P. putida* cells and observed that the fluorescence signal was increased in the CrcB-deficient strain when BenE-I was also deleted. However, the complementation of this double mutant with the *P_tac_-benE-I* expression cassette did not reduce the fluorescence signal. Our results also revealed that elevated fluoride concentration inside cells was associated with compromised membranes, as the population of cells with propidium iodide-permeable cells was remarkably increased in the presence of NaF (Fig. 8). Thus, our results rather imply that BenE-I contributes to fluoride tolerance through an indirect mechanism. Previous studies (14) demonstrated that *P. putida* CrcB-deficient mutant suffers acidification of the cytoplasm when bacteria are exposed to fluoride. This study also indicated that fluoride exerts a multi-level stress response on *P. putida*, which affects central carbon metabolism and energy metabolism. Importantly, many of the actions of fluoride are related to those of organic weak acids, including metabolic acids, fatty acids, and benzoic acid—all of which de-energize the cell membrane (26). Given that BenE-I is characterized as one of the benzoate transporters in *P. putida*, this protein could somehow influence both benzoate and fluoride stress responses. Thus, BenE-I could act not only as a substrate-specific transporter of benzoate but also as a structural and regulatory component of the membrane stress response by influencing fluoride tolerance independently of benzoate.

What the operon *PP_2034-PP_2037*, where *benE-I* belongs, is unclear, as the aldolase and 4-hydroxy-tetrahydrodipicolinate synthase belong to different metabolic pathways and different steps, and all three other genes have no pathways associated with them. Based on the KEGG database, aldolase PP_2037 is predicted to belong to six different pathways: monobactam biosynthesis, lysine biosynthesis, metabolic pathways, biosynthesis of secondary metabolites, microbial metabolism in diverse environments, and biosynthesis of amino acids; 4-hydroxy-tetrahydrodipicolinate synthase is predicted to belong to the following four different pathways: ppu00040 Pentose and glucuronate interconversions, fructose and mannose metabolism, metabolic pathways, and microbial metabolism in diverse environments (30; entry ppu:PP_2036: available from: https://www.kegg.jp/entry/ppu:PP_2036; retrieved 23 March 2026; entry ppu:PP_2037: available from: https://www.kegg.jp/entry/ppu:PP_2037; retrieved 23 March 2026).

The lack of clear functional linkage among genes in the *PP_2034–PP_2037* operon raises the possibility that their roles in fluoride tolerance may not be confined to canonical metabolic pathways. Instead, they could participate in alternative processes through functional versatility. One intriguing explanation is the phenomenon of protein moonlighting, where a single protein carries out more than one unrelated role. Such multifunctionality has been increasingly recognized in bacteria, where metabolic enzymes, structural proteins, and transporters adopt additional roles beyond their canonical functions (31, 32). For example, enolase and glyceraldehyde-3-phosphate dehydrogenase are well-known metabolic enzymes that also act as RNA-binding proteins or surface adhesins in diverse bacterial species (33, 34). In the context of fluoride tolerance, the possibility that BenE-I exhibits moonlighting activities cannot be excluded. Given the observed regulatory complexity and the lack of clear pathway associations for most genes in this operon, it is plausible that their encoded proteins may perform secondary functions relevant to ion homeostasis, membrane remodeling, or stress adaptation.

The regulatory landscape of PP_3125 may extend beyond what was captured in our proteomic and transcriptomic snapshots, which were limited to early growth phases. It is likely that PP_3125 influences additional gene sets during later growth stages, potentially coordinating broader adaptive responses to prolonged fluoride exposure.

Interestingly, spontaneous fluoride-tolerant mutants showed large genomic deletions encompassing *PP_3125* and up to 344 other genes. These deletions varied in size, from 18 to 345 genes, and included ECF family sigma factors, DNA polymerases (e.g., *dnaEB, imuB*), transporters, regulators, hydrolases, and multiple catabolic pathways such as benzoate and phenylacetate degradation operons (Table S1). Despite these extensive deletions, the mutants exhibited no significant growth defects under standard laboratory conditions and showed improved growth under fluoride stress (Fig. 4), suggesting that the deleted regions may include nonessential genes under those conditions or that the deletions conferred adaptive benefits.

Multiple mechanisms may underlie these large-scale deletions. Genome plasticity in *P. putida* is supported by the presence of mobile genetic elements such as transposons and prophages. For instance, the *bph-sal* element in *P. putida* KF715 is known to be both deletable and transferable, reflecting high genomic fluidity (35). Moreover, *P. putida* KT2440 contains prophages capable of spontaneous excision, which may promote deletion events even in the absence of phage infectivity (36). It is possible that environmental stresses like fluoride exposure can cause DNA damage, and imprecise repair via the non-homologous end joining (NHEJ) pathway (37) may result in random deletions, but as the deletions are 18–300 kb, there needs to be other mechanisms involved too. The diversity in the start and end points of observed deletions (Fig. S1) further suggests the absence of a single deletion mechanism and highlights the stochastic nature of stress-induced genomic rearrangements.

Taken together, our data support a model in which PP_3125 functions as a central negative regulator of fluoride tolerance, suppressing the expression of transporter genes like *benE-I* that mitigate fluoride toxicity. Deletion of *PP_3125*, whether through spontaneous genomic rearrangement or targeted mutagenesis, leads to upregulation of these protective pathways. The fluoride-responsive expression pattern of PP_3125 hints at a yet-to-be-characterized regulatory mechanism that senses and responds to fluoride stress.

In conclusion, this study identifies PP_3125 as a key node in the regulation of fluoride tolerance in *P. putida* and highlights BenE-I as a promising target for engineering enhanced fluoride tolerance. The evolutionary emergence of large deletions encompassing PP_3125 under stress conditions reflects the organism’s remarkable genomic plasticity. Future studies should elucidate the precise function of BenE-I in fluoride tolerance, the broader regulon of PP_3125 across growth phases, and the molecular triggers underlying stress-induced genomic deletions.

## MATERIALS AND METHODS

### Bacterial strains, plasmids, and culture media

Bacterial strains and plasmids used in this study are listed in Table 1. *P. putida* strains are derivatives of KT2440 (38). Bacteria were grown in lysogeny broth (LB) or a minimal medium with M9 buffer, where casamino acids (CAA) and glucose were added at final concentrations of 0.2. *E. coli* was incubated at 37°C, and *P. putida* at 30°C. When selection was needed, the growth medium was supplemented with antibiotics at the following concentrations: kanamycin 50 μg · mL^−1^, ampicillin 100 μg · mL^−1^, streptomycin 200 μg · mL^−1^, and gentamycin 10 μg · mL^−1^. Bacteria were electrotransformed according to the protocol described by Sharma and Schimke (39). Sodium fluoride (NaF) was purchased from Sigma-Aldrich (St. Louis, MO, USA; cat. #201154) and used at concentrations ranging from 0 to 30 mM, as specified in the text.

**TABLE 1:** Strains and plasmids used in this study

Bacterial strain or plasmid	Relevant characteristics	Source or reference
*E. coli*		
DH5α λ*pir*	Cloning host; F^−^λ^−^*endA1 glnX44*(AS) *thiE1 recA1 relA1 spoT1 gyrA96*(Nal^R^) *rfbC1 deoR nupG* Φ80(*lacZ*Δ*M15*) Δ(*argF-lac*)*U169 hsdR17*(*r_K_*^−^*m_K_*^+^), λ*pir* lysogen	(40)
CC118 λpir	Cloning host; Δ(*ara-leu*) *araD* Δ*lacX174 galE galK phoA thiE1 rpsE rpoB*(Rif^R^) *argE*(Am) *recA1*, λ*pir* lysogen	(41)
HB101	Helper strain; F^−^λ^−^*hsdS20*(*r_B_*^−^*m_B_*^−^) *recA13 leuB6*(Am) *araC14* Δ(*gpt-proA*)*62 lacY1 galK2*(Oc) *xyl-5 mtl-1 thiE1 rpsL20*(Sm^R^) *glnX44*(AS)	(42)
*P. putida*		
KT2440	Wild-type strain; mt-2 derivative cured of the TOL plasmid pWW0	(38)
Δ*crcB*	KT2440 *∆crcB*	(7)
Δ*crcB*Δ*3125*	KT2440 ∆*crcB* Δ*PP_3125*	This study
Δ*3125*	KT2440 ∆*PP_3125*	This study
Δ*crcB*Δ*3125*_tac_*3125*	KT2440 Δ*crcB*Δ*3125* strain containing *lacI*-P_tac_-*PP_3125* in the intergenic region between *glmS* and PP5408 (Gm^r^)	This study
Δ*crcB*Δ5	KT2440 ∆*crcB* Δ*PP_2033*, Δ*PP_2034*, Δ*benE-I*, Δ*PP_2036* Δ*PP_2037*	This study
*crcB1*	Spontaneous NaF^+^ Δ*crcB* mutant with genomic deletion [*PP_2985*]-*PP_5598*	This study
*crcB4*	Spontaneous NaF^+^ Δ*crcB* mutant with genomic deletion [*PP_2985*]-[*PP_3288*]	This study
*crcB12*	Spontaneous NaF^+^ Δ*crcB* mutant with genomic deletion [*PP_3119*]-[*PP_3133*]	This study
*crcB18*	Spontaneous NaF^+^ Δ*crcB* mutant with genomic deletion [*PP_3059*]-[*PP_3221*]	This study
*ΔcrcBΔbenE-I*	KT2440 ∆*crcB* Δ*benE-I*	This study
Δ*crcB*Δ*benE-I*_tac_*benE-I*	KT2440 *ΔcrcBΔbenE-I* strain containing *lacI*-P_tac_-*benE-I* in the intergenic region between *glmS* and PP5408 (Gm^r^)	This study
Δ*crcB*Δ*3125*Δ*ald*	KT2440 ∆*crcB* Δ*ald* (*PP_2037*)	This study
Δ*crcB*Δ*3125*Δ*ald*_tac_ald	KT2440 *ΔcrcBΔbenE-I* strain containing *lacI*-P_tac_-*ald* in the intergenic region between *glmS* and PP5408 (Gm^r^)	This study
Plasmids		
pBAM1	Delivery plasmid for mini-Tn5 (Amp^r^, Km^r^)	(43)
pRK2013	Helper plasmid for conjugal transfer (Km^r^)	(44)
pSEVA/lacItac	Broad-host range plasmid containing *lacI*-P_tac_ cassette (Km^r^)	(45)
pSNW2	pEMG derivative with P14g(BCD2)→msfGFP (Km^r^)	(46)
pSW(I-SceI)	Plasmid for I-SceI expression (Amp^r^)	(47)
pSNW2_*3125*_before	pSNW2 plasmid containing a fragment of 500 nucleotides upstream of the *PP_3125* gene with BamHI and SacI restriction sites (Km^r^)	This study
pSNW2_*3125*_before+after	pSNW2 plasmid containing fragments of 500 nucleotides upstream and 500 nucleotides downstream of the *PP_3125* gene, with a SacI restriction site between the two fragments (Km^r^)	This study
pSEVA/lacItac-*3125*	pSEVA/lacItac containing gene cassette *lacI*-P_tac_-*PP_3125* (Km^r^)	This study
pGP704LTn7GmlacItac-*3125*	pGP-miniTn7-ΩGm containing gene cassette *lacI*-P_tac_-*PP_3125* between inverted repeats of mini-Tn7 (Amp^r^, Gm^r^)	This study
pGP704LTn7GmlacItac-*benE-I*	pGP-miniTn7-ΩGm containing gene cassette *lacI*-P_tac_-*benE-I* between inverted repeats of mini-Tn7 (Amp^r^, Gm^r^)	This study
pGP704LTn7GmlacItac-*ald*	pGP-miniTn7-ΩGm containing gene cassette *lacI*-P_tac_-*ald* (*PP_2037*) between inverted repeats of mini-Tn7 (Amp^r^, Gm^r^)	This study
pSNW2/del5	pSNW2 plasmid containing fragments of 500 nucleotides after *PP_2033* and 500 nucleotides after *PP_2037* gene (Km^r^)	This study
pSNW2/del_*benE-I*	pSNW2 plasmid containing fragments of 500 nucleotides upstream and downstream of the *benE-I* (Km^r^)	This study
pSNW2/del_ald	pSNW2 plasmid containing fragments of 500 nucleotides upstream and downstream of the aldolase *PP_2037* (Km^r^)	This study
pSEVA/lacItac-*benE-I*	pSEVA/lacItac containing gene cassette *lacI*-P_tac_-*benE-I* (Km^r^)	This study
pSEVA/lacItac-*2034*	pSEVA/lacItac containing gene cassette *lacI*-P_tac_-*PP_2034* (Km^r^)	This study
pSEVA/lacItac-*ald*	pSEVA/lacItac containing gene cassette *lacI*-P_tac_-*ald* (*PP_2037*) (Km^r^)	This study
pGP-miniTn7-ΩGm	pGP704 L carrying a SacI–XbaI mini-Tn7-ΩGm cassette from pBK-miniTn7-ΩGm (Ampr, Gm^r^)	(48)
pUX-BF13	Helper plasmid, providing the Tn*7* transposase proteins (Amp^r^ *mob+*)	(49)
pS441·FRSv1	Plasmid-borne fluoride biosensor based on a fluoride-responsive riboswitch (FRS); derivative of vector pSEVA441, FRSv1 → *msfGFP*; Str^R^	(7)

### Construction of plasmids and strains

Oligonucleotides used in this study are listed in Table 2. For the construction of the *PP_3125*-deficient strain, 500 nucleotides of the upstream and downstream region of *PP_3125* were amplified from chromosomal DNA of *P. putida* KT2440 with oligonucleotide*s* PP_3125_BHI and PP_3125delSac for the upstream region and PP_3125outdelSac and *PP_3125*_EcoRI for the downstream region. The PCR product of the upstream region of PP_3125 was cloned into BamHI- and SacI-opened pSNW2, resulting in pSNW2_*3125*_before. Next, the pSNW2_*3125*_before was opened with SacI and EcoRI, and the downstream region of *PP_3125* was cloned into the vector, resulting in the vector pSNW2_*3125*_before+after. A combination of previously published methods was used to delete genes in *P. putida* strains (46, 47). The plasmid pSNW2_*3125*_before+after was electroporated into *P. putida* KT2440 WT and Δ*crcB* strains. Kanamycin-resistant colonies with visible GFP expression under blue light, carrying a cointegrate in the chromosome, were isolated on kanamycin selective plates and then were electroporated with the I-SceI expression plasmid pSW(I-SceI). To resolve cointegrates, the plasmid-encoded I-SceI nuclease was induced by cultivating bacteria overnight in LB medium supplemented with 1.5 mM 3-methylbenzoate. Kanamycin-sensitive colonies were isolated, and PCR and DNA sequencing verified the PP_3125 deletion. The deletion strains were cured of the plasmid pSW(I-SceI) by overnight growth without antibiotics. The absence of pSW(I-SceI) was confirmed by PCR.

**TABLE 2:** Primers used in this study^*a*^

Primer	Sequence (5'−3')	Description/Use
T1T2	GGCCTTTTTGCGTAGATC	For verification of fragment insertion into the pSEVA/lacItac plasmid
ARB6	GGCCACGCGTCGACTAGTACNNNNNNNNNNACGCC	Primer for the first reaction of ARB-PCR
BAM1	TTATGTAAGCAGACAGTTTT	Primer for the first reaction of ARB-PCR, complementary to the region in the Tn5 mini-transposon
ARB2	GGCCACGCGTCGACTAGTAC	Primer for the second reaction of ARB-PCR, complementary to the primer from the first reaction
Me-I-uus2	TATCTTGTGCAATGTAACATCAGAG	For the second reaction of ARB-PCR, complementary to the region in the Tn5 mini-transposon
PP_3125_BHI	TAGGATCCTCACCCAGGTGAAGGGC	For the *PP_3125* deletion strain
PP_3125delSac	ATAGAGCTCATGTGACGCGAGTT	For the *PP_3125* deletion strain
PP_3125outdelSac	TAAGAGCTCTGAAAGCGCAAGGGGGCA	For the *PP_3125* deletion strain
PP_3125_EcoRI	GAGAATTCACAAGCCACGCCTG	For the *PP_3125* deletion strain
PP_3125algHIII	TATAAGCTTATGTCTATCCGATTGAAACTG	For the *PP_3125* overexpression strain
PP_3125loppSalI	TATGTCGACTCATTCGTCTGCGTGGTGAAC	For the *PP_3125* overexpression strain
BenE-I_start_HIII	TATAAGCTTATGGACGCGCCTGCTCAAAG	For constructing the *benE-I* gene overexpression, construct
BenE-I_stop_Xba	ATATCTAGATTAGCGGGAGGCGCCG	For constructing the *benE-I* gene overexpression, construct
PP_2034_start_HIII	TATAAGCTTGCCGTGCGCCAGGG	*PP_2034* overexpression construct
PP_2034_stop_Xba	ATATCTAGACTACAG GGCGCGCGCCAGCT	*PP_2034* overexpression construct
5_del	GACAAGCAGGAGACACGATGTCATGACATAGGCGGTACCTGCAGGGGG	For *PP_2033 - PP_2037* deletion strain
5_out	TCATGACATCGTGTCTCCTGCTTGTC	For *PP_2033 - PP_2037* deletion strain
2033_EcoRI	AATGAATTCACGAAGCTGCCGGTTTC	For *PP_2033 - PP_2037* deletion strain
PP_2037-BHI	TAGGATCCCAGGCGCGCATTGACCGGTAC	For *PP_2033 - PP_2037* deletion strain and for the *PP_2037*, aldolase deletion strain
BenE-I_BHI	TAGGATCCGCCTGTTCACGGCTAC	For the *benE-I* deletion strain
BenE-I_del	GTGGTGGAAAGCTCGCTTACCATGGTTGCCTCTGGTAATG	For the *benE-I* deletion strain
BenE-I_out	TAAGCGAGCTTTCCACCAC	For the *benE-I* deletion strain
BenE-I_EcoRI	AATGAATTCGGCCGAACCGAGGAAAC	For the *benE-I* deletion strain
BenE-I_start_HIII	TATAAGCTTATGGACGCGCCTGCTCAAAG	For the *benE-I* overexpression strain
BenE-I_stop_Xba	ATATCTAGATTAGCGGGAGGCGCCG	For the *benE-I* overexpression strain
PP_2037_out	TGATCGAAGCTGTACTCTTTCC	For the *PP_2037*, aldolase deletion strain
PP_2037_EcoRI	AATGAATTCCGGGTTGTTGTAGAGCATGATCGG	For the *PP_2037*, aldolase deletion strain
PP_2037del	GGAAAGAGTACAGCTTCGATCACATAGGCGGTACCTGCAGGGGG	For the *PP_2037*, aldolase deletion strain
PP_2037 start-HIII	TATAAGCTTATGACGACCACCATGCACACCCC	For the *PP_2037*, aldolase overexpression strain
2037_stop_XbaI	ATATCTAGACCGTTGTTGAGGAAAGAGTACAGCTTCGATCA	For the *PP_2037*, aldolase overexpression strain

^*a*^ The sites for restriction enzymes are underlined.

For the construction of the *PP_2033–PP_2037* operon-deletion strain, aldolase *PP_2037,* and the *benE-I* deletion strain, a combination of previously published methods (46, 47) was used to generate a deletion with scar as suggested by Wirth et al., (50). Approximately 500 bp from the upstream and downstream regions of the genomic DNA intended to be deleted were separately amplified and then fused into one (∼1 kb) DNA fragment by overlap extension PCR, which was followed by restriction cloning into the plasmid pSNW2. A set of pSNW2-based plasmids generated is listed in Table 1, and primers are listed in Table 2.

For the construction of overexpression constructs, the genes *PP_3125, PP_2034, PP_2037* (aldolase), and *benE-I* were amplified by PCR from the chromosome and inserted into the vector plasmid pSEVA/lacItac by using the HindIII and SalI restriction sites for *PP_3125*, and HindIII and XbaI for *PP_2034* and *benE-I,* resulting in pSEVA/lacItac-*3125,* pSEVA/lacItac-*benE-I,* pSEVA/lacItac-*ald,* or pSEVA/lacItac-*2034*, respectively. To construct the overexpression strains of *PP_3215, PP_2037,* and *benE-I,* the whole gene cassette from the pSEVA/lacItac construct was cloned into the pGP-miniTn7-ΩGm (48) vector as a NotI fragment, resulting in plasmid pGP704LTn7GmlacItac-*3125,* pGP704LTn7GmlacItac-*ald,* or pGP704LTn7GmlacItac*benE-I*. All previous steps were carried out in *E. coli*. To deliver the gene cassette to the Tn7 insertion site in the chromosome of *P. putida,* the previously published method was used (51). *P. putida* strains were co-electroporated with a plasmid carrying the overexpression cassette and helper plasmid pUX-BF13 (49). The positive clones on LB Gm plates were identified by PCR and verified by sequencing.

### Estimation of the frequency of spontaneous NaF-tolerant mutants in the *ΔcrcB* strain

To analyze the occurrence of spontaneous NaF-tolerant mutants in the Δ*crcB* strain, bacteria were grown for 6 h in LB liquid medium, a 10^−5^ dilution of the cultures was made, dispensed into at least 10 test tubes as 2.3 mL aliquots, and allowed to reach saturation by growing cells for 18 h; ~3 × 10^9^ cells were plated onto LB plates containing 5 mM NaF and incubated at 30 °C. The NaF^+^ colonies were counted on day 5. The frequency of NaF^+^ colonies is the number of NaF^+^ colonies per milliliter divided by CFU. Four biological replicates with at least 10 parallels were analyzed.

### Whole genome sequencing

For whole genome sequencing, DNA from spontaneous NaF+ Δ*crcB* mutants was extracted using the Thermo Scientific GeneJET Genomic DNA Purification Kit. Sequencing was performed on the Illumina MiSeq platform with 100× coverage at the University of Tartu. All the resulting raw sequences are available in GenBank under BioProject ID PRJNA1330567. The received raw data were trimmed using Trimmomatic ver. 0.39 (52). Mutations in the sequences were detected using Breseq version 0.36.0 (53). Changes in the chromosome are mapped against the published genome of *P. putida* KT2440 (15).

### Transposon mutagenesis and selection of transposon mutants

A transposon mutagenesis of *P. putida* Δ*crcB* was performed to identify the genes affecting fluoride tolerance. Mini-transposon Tn5 (Km^r^)-carrying plasmid pBAM1 (43) replicating only in *E. coli* hosts containing the phage lambda *pir* gene (e.g., *E. coli* DH5α λ*pir*) was transferred into the Δ*crcB* by conjugation with the aid of the helper plasmid pRK2013 (44). Transconjugants of Δ*crcB* carrying random insertions of mini-Tn5 in the chromosome were selected on LB-Km plates. The total number of transposon mutant colonies was counted, and the colonies were transferred to LB plates containing 15 mM NaF using replica plating. About 141,000 transposon mutants from three independent transposon mutagenesis experiments were obtained, out of which 48 had a fluoride-tolerant phenotype. The NaF-tolerant colonies were isolated and subjected to the determination of the location of the mini-Tn5 insertion in the chromosome by arbitrary PCR and DNA sequencing.

### Arbitrary PCR

To identify the mini-Tn5 insertion site in the chromosome of *P. putida* Δ*crcB* transposon mutants, an arbitrary PCR and DNA sequencing were performed. The arbitrary PCR products were obtained according to a published protocol (54) by two rounds of amplification. In the first round of PCR, primers BAM1 and arbitrary primer ARB6 were used. The second-round PCR product was generated with primers ME-I-uus2 and arbitrary primer ARB2. DNA sequencing of the PCR products was performed using the BigDye Terminator v3.1 Cycle Terminator kit and analyzed with the Applied Biosystems 3730xl DNA sequencer.

### Growth analysis experiments

For growth analysis experiments, the cells were grown overnight in liquid LB medium and then diluted to an OD600 of 0.05, and NaF was added at a final concentration ranging from 0 mM to 2.5 mM before transferring 100 µL of the diluted culture to the 96-well plate. The optical density was measured automatically every 10 min for 24 h (96 flat-bottom; Greiner Bio-One, Kremsmünster, Austria). Data were analyzed, and graphs and growth measurements were obtained from a QurvE program version 1.1 (55). Statistical analysis was done by using GraphPad Prism 10.0. D′Agostino & Pearson test was used to determine the normality of the data set. As the data did not follow a normal distribution, the non-parametric method was used to compare different data sets. A Kruskal-Wallis test, followed by Dunn’s post-hoc test, was performed. Cell growth on solid medium was evaluated using dilution spot assays. Bacteria were grown overnight in 5 mL of LB-Km medium; 10-fold serial dilutions of the cultures were spotted as 5 µL drops onto LB-Km agar plates supplemented with 0.5 mM IPTG and different concentrations of NaF (0–30 mM NaF). Plates were incubated at 30°C for 20 h. Two technical replicate plates were done for each NaF concentration.

### Proteomic and transcriptomic analyses

Proteomic and transcriptomic samples were prepared from cultures grown in the same conditions. For proteomic analysis, three, and for the transcriptomic analysis, two independent cultures of each *P. putida* strain were sent to be analyzed. As the lag phase is the growth parameter most affected by the fluoride stress (27), to obtain similar effects, all strains were grown in different NaF concentrations that had a similar impact on lag phase (the lag phase was two times longer than without NaF). For the Δ*crcB* strain, 0.2 mM NaF was used; for the Δ*crcB*Δ*3125* strain, 3.5 mM NaF was used; for the Δ*3125* strain, 30 mM NaF was used; and for the WT strain, 33 mM NaF was used. Cells were grown overnight and diluted to fresh media to an OD_580_ of 0.05. Cultures were grown in biological duplicates until the beginning of the exponential phase, with an OD_580_ of 0.1, and biomass was harvested and washed twice with 1× M9 buffer (42 mM KH2PO4, 24 mM Na2HPO4, 19 mM NH4Cl, and 9 mM NaCl). Cell pellets corresponding to 1 mL of OD580 = 1 were flash-frozen in liquid nitrogen and stored at −80°C until further use.

For proteomics, label-free quantification of the whole-cell proteome was performed by LC-MS/MS with LTQ-Orbitrap XL (Thermo Fisher Scientific) coupled to an Agilent 1200 nanoflow LC via nanoelectrospray ion source (Proxeon) in the Proteomics Core Facility, Institute of Technology, University of Tartu, Estonia. The data were analyzed using MaxQuant and Perseus software (Max Planck Institute of Biochemistry, Planegg, Germany) (56). Parallel samples were grouped, and groups were compared in pairs: deletion strains against WT strains and parallels with NaF against parallels without NaF. To be included in the analysis, a protein must be detected in all three replicates of one group. Thereafter, missing values were imputed using default settings. Mean protein abundances were compared between the two groups using the independent-sample Student *t*-test. The Benjamini-Hochberg multiple-testing correction was applied with the false discovery rate set to 0.05. The mass spectrometry proteomics data have been deposited to the ProteomeXchange Consortium via the PRIDE (57) partner repository with the data set identifier PXD068884.

For transcriptomics, the cultures were prepared the same way as for the proteomic analysis. RNA extraction and purification were performed by the Global Genomic Center in Hong Kong, China. RNA-seq analysis was performed to investigate transcriptomic responses under NaF toxicity conditions. Raw sequencing data were processed using the DESeq2 package (v1.48.1) within R (v4.5.1), applying standard pipelines for normalization, differential expression analysis, and statistical significance testing. Differentially expressed genes were determined using DESeq2’s default parameters, with *P*-values adjusted via the Benjamini-Hochberg method to control for false discovery rate. The R code employed for data visualisation and enhanced customisation of principal component analysis (PCA), MA plots, and volcano plots has been made available at https://github.com/puiggene07/PubSuppl/tree/main/2026_NaF_tolerance_P_putida. The data have been deposited in NCBI’s Gene Expression Omnibus (58) and are accessible through GEO Series accession number GSE309641.

### Assessing the intracellular F^−^ concentration with a fluoride-responsive fluorescent biosensor

Plasmid pS441·FRSv1 (7), carrying a monomeric superfolder GFP (msfGFP) gene under the control of a synthetic riboswitch-promoter element, was transformed into the wild-type strain and the *P. putida* Δ*crcB*, Δ*crcB*Δ*benE-I*, and Δ*crcB*Δ*benE-I*_tac_*benE-I* strains. The intracellular F^−^ concentration in these strains correlated with msfGFP fluorescence. The strains carrying plasmid pS441·FRSv1 were grown overnight in M9 minimal medium with streptomycin. Overnight pre-cultures were diluted 20-fold into 4 mL of M9 minimal medium, and the cell suspension was distributed into 96-well microtiter plates (uClear Black flat-bottom; Greiner Bio-One, Kremsmünster, Austria). Cells were grown for 3 h at 30°C with agitation at 282 rpm, after which NaF was added at 0, 0.25, and 1 mM concentrations. The kinetics of bacterial growth (estimated by OD_580_) and msfGFP fluorescence (excitation and emission wavelengths of 485 and 528 nm, respectively) were monitored for 20 h. Measurements were carried out every 10 min on three independent biological replicates, and the normalized fluorescence values at 7, 10, and 15 h after NaF addition were plotted and compared across conditions using GraphPad Prism 10.0.

### Flow cytometry analysis

Bacteria were grown in the same conditions as for the fluoride biosensor experiment. The cells from the 15-h time point were diluted into M9 buffer to an OD_580_ of 0.02. The two components of the LIVE/DEAD BacLight kit (L7012; Invitrogen), 20 mM red fluorescent dye propidium iodide (PI) and 3.34 mM green fluorescent dye SYTO9, were mixed at a 1:1 volume ratio and then diluted 17.6-fold into filter-sterilized M9 buffer. For staining bacteria, the diluted cell suspension was mixed with a freshly prepared reagent mixture at a 1:20 ratio. Samples were incubated at room temperature in the dark for 30 min, and approximately 10,000 events from each sample were analyzed using a FACSAria flow cytometer (BD Biosciences). Fluorescent dyes were excited at 488 nm. Forward and side scatter of the light and fluorescence emission at 530 and 616 nm were acquired for every event. FlowJo software was used for data analysis. Populations of intact and PI-permeable cells were defined as previously described (59). Two biological replicas in two technical parallels were analyzed.

## Data Availability

Further information supporting the findings of this study is available upon request from the corresponding author. All the resulting raw sequences from whole genome sequencing are available in GenBank under BioProject ID PRJNA1330567. The mass spectrometry proteomics data have been deposited to the ProteomeXchange Consortium via the PRIDE (59) partner repository with the data set identifier PXD068884. The transcriptomics data have been deposited in NCBI’s Gene Expression Omnibus (58) and are accessible through GEO Series accession number GSE309641.

## SUPPLEMENTAL MATERIAL

Supplemental tables and figures jb.00479-25-s0001.pdf Tables S1 and S2; Figures S1 to S4.

Table S3 jb.00479-25-s0002.xlsx Transcriptome results.

ASM does not own the copyrights to Supplemental Material that may be linked to, or accessed through, an article. The authors have granted ASM a non-exclusive, world-wide license to publish the Supplemental Material files. Please contact the corresponding author directly for reuse.
