# Supplementary material for: Adaptive evolution of Pseudomonas putida in the presence of fluoride exposes novel functions of a benzoate transporter
Source: J Bacteriol. 2026 Apr 1;208(4):e00479-25. doi: 10.1128/jb.00479-25 (PMC13104614; doi:10.1128/jb.00479-25)
Supplement: Supplemental tables and figures — Tables S1 and S2; Figures S1 to S4. [file jb.00479-25-s0001.pdf]

Table S1. Deleted genes in the spontaneous NaF[+] *ΔcrcB* mutant (*ΔcrcB-12)*

Gene number	Gene name	Function
PP_3119	*dnaEB*	error-prone DNA polymerase
PP_3120	*yeaE*	methylglyoxal reductase
PP_3121		transcriptional regulator, LysR family
PP_3122	*atoA*	3-oxoacid CoA-transferase subunit A
PP_3123	*atoB*	3-oxoacid CoA-transferase subunit B
PP_3124		short chain fatty acid transporter
PP_3125		Cro/CI family transcriptional regulator
PP_5586		hypothetical protein
PP_3126		polysaccharide biosynthesis/export protein
PP_5587		hypothetical protein
PP_3127		exopolysaccharide transport protein
PP_3128		exopolysaccharide biosynthesis/transport protein
PP_3129	*galE*	UDP-glucose 4-epimerase
PP_3130		hypothetical protein
PP_3131		membrane protein
PP_3132		polysaccharide transporter
PP_5588		hypothetical protein
PP_3133		oxidoreductase

Table S2. Differently expressed genes from transcriptomic analysis in *P. putida* Δ*3125* strain compared to WT strain in the presence of NaF.

**locus tag**	**product**	
PP_1277	mannose-1-phosphate guanylyltransferase/mannose-6-phosphate isomerase	slightly downregulated also in the presence of NaF in the Δ*crcB* strain, probably the fluoride tolerance effect
PP_1278	alginate O-acetyltransferase AlgF	slightly downregulated also in the presence of NaF in the Δ*crcB* strain, probably the fluoride tolerance effect
PP_1279	alginate O-acetyltransferase	slightly downregulated also in the presence of NaF in the Δ*crcB* strain, probably the fluoride tolerance effect
PP_1280	MBOAT family protein	slightly downregulated also in the presence of NaF in the Δ*crcB* strain, probably the fluoride tolerance effect
PP_1281	mannuronate-specific alginate lyase	slightly downregulated also in the presence of NaF in the Δ*crcB* strain, probably the fluoride tolerance effect
PP_1282	alginate O-acetyltransferase	slightly downregulated also in the presence of NaF in the Δ*crcB* strain, probably the fluoride tolerance effect
PP_1284	alginate export family protein	slightly downregulated also in the presence of NaF in the Δ*crcB* strain, probably the fluoride tolerance effect
PP_1286	PilZ domain-containing protein	slightly downregulated also in the presence of NaF in the Δ*crcB* strain, probably the fluoride tolerance effect
PP_1287	glycosyltransferase family 2 protein	slightly downregulated also in the presence of NaF in the Δ*crcB* strain, probably the fluoride tolerance effect
PP_1288	UDP-glucose/GDP-mannose dehydrogenase family protein	slightly downregulated also in the presence of NaF in the Δ*crcB* strain, probably the fluoride tolerance effect
PP_3542	DUF3203 family protein	downregulated even more *crcB* is deleted, probably the fluoride tolerance effect
PP_RS21265	hypothetical protein	slightly upregulated also in the presence of NaF in the Δ*crcB* strain, probably the fluoride tolerance effect

PP_2033	DUF2235 domain-containing protein	overexpressed only when PP_3125 is deleted
PP_2034	MFS transporter	overexpressed only when PP_3125 is deleted
PP_2035	benzoate/H(+) symporter BenE family transporter	overexpressed only when PP_3125 is deleted
PP_2036	dihydrodipicolinate synthase family protein	overexpressed only when PP_3125 is deleted
PP_2037	aldolase	overexpressed only when PP_3125 is deleted
PP_0608	prepilin-type N-terminal cleavage/methylation domain-containing protein	upregulated also in the presence of NaF in the Δ*crcB* strain, probably the fluoride tolerance effect
PP_1729	hypothetical protein	upregulated also in the presence of NaF in the Δ*crcB* strain, probably the fluoride tolerance effect

Figure S1. Spontaneous NaF-tolerant Δ*crcB* mutants crcB1, crcB4, crcB12, and crcB18 with their respective genomic deletions (marked with different colours). The different genomic deletions are shown with the isolate number and the deletion size in kilobases.

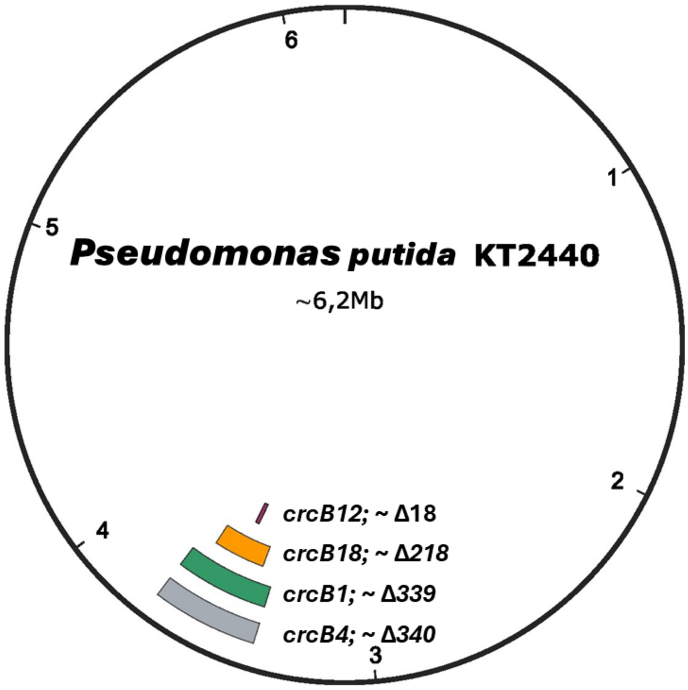

Figure S2. Volcano plot showing results of proteomic analysis where the Δ*crcB*Δ*3125* strain is compared to the WT strain. All strains had three technical replicates. The red line shows results with statistically significant differences. The black line shows results expressed over or down 100 times, the green line 10000 times, as the difference is logarithmic during analysis.

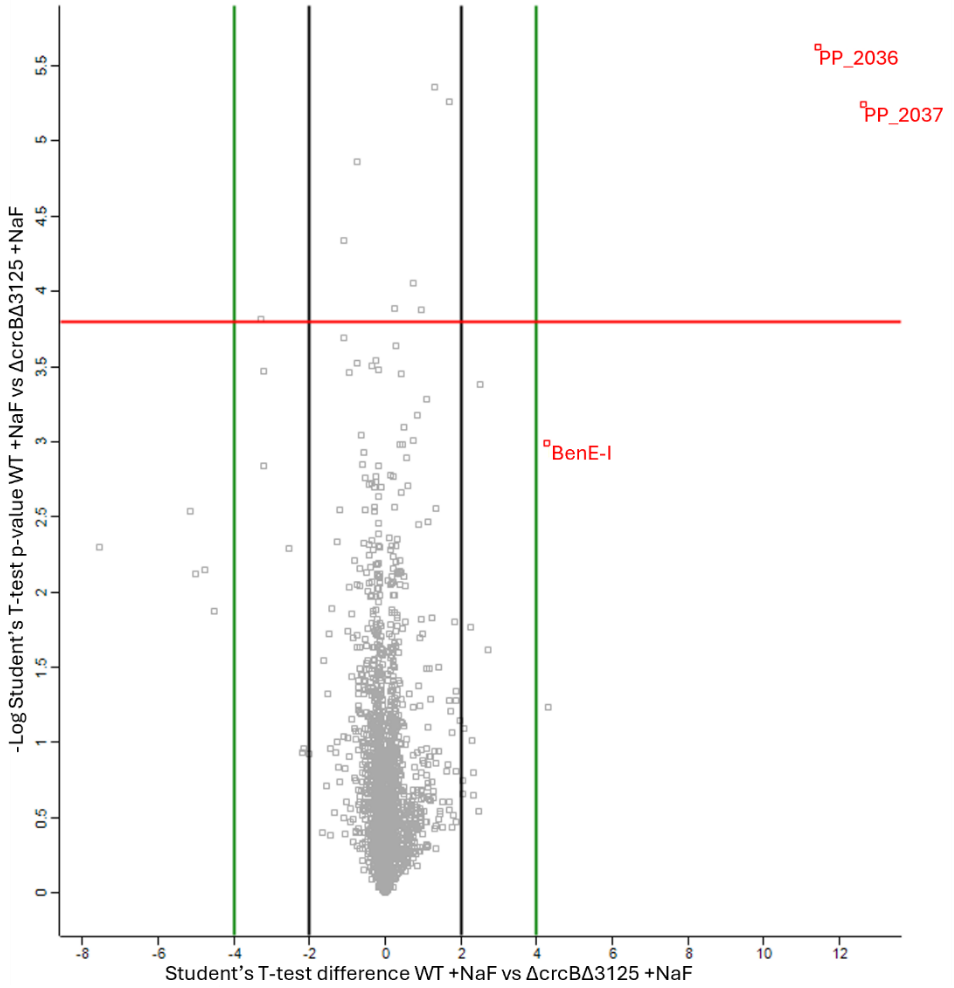

Figure S3. Volcano plot showing results of proteomic analysis where the Δ*crcB*Δ*3125* strain is compared to the Δ*crcB* strain. All strains had three technical replicates. The red line shows results with statistically significant differences. The black line shows results expressed over or down 100 times, the green line 10000 times, as the difference is logarithmic during analysis.

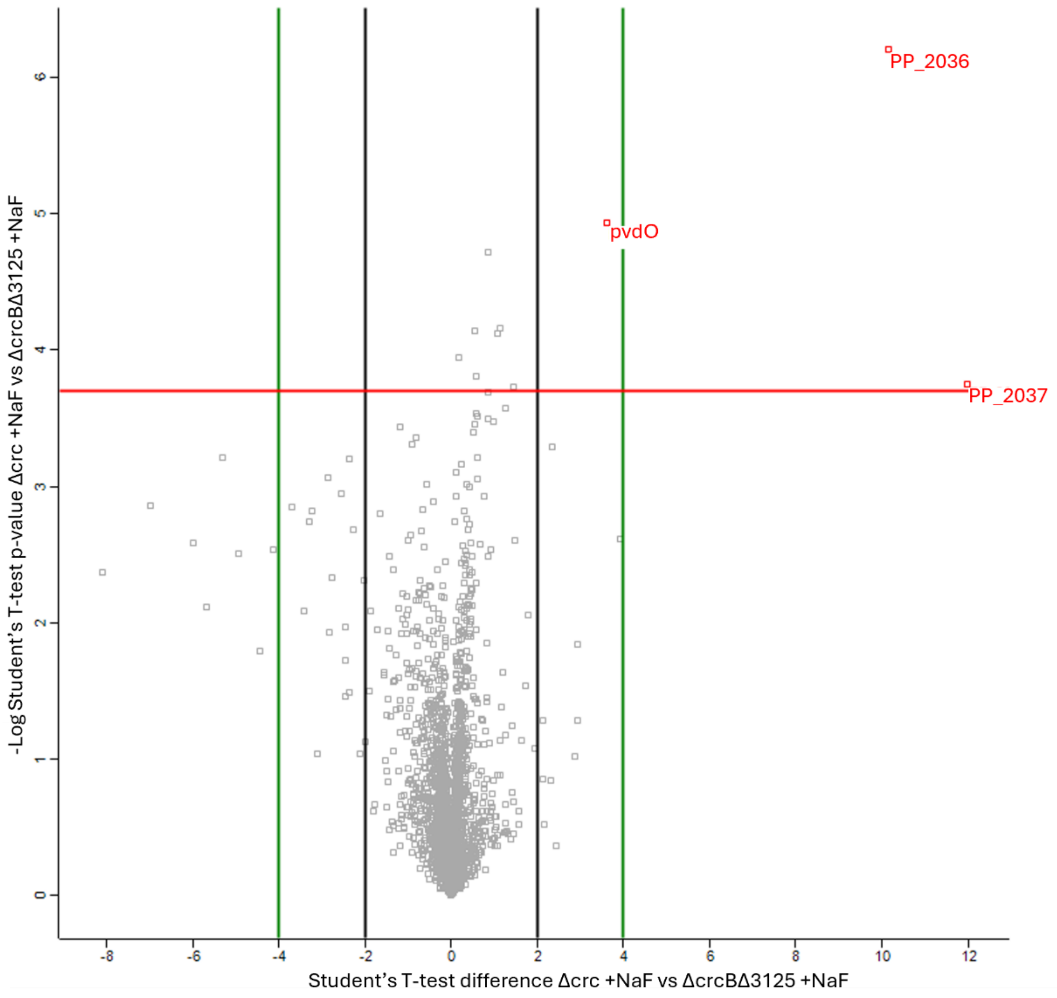

Figure S4. Growth of *P. putida* Δ*crcB*, Δ*crcB*Δ*3125*Δ*ald,* and Δ*crcB*Δ*3125*Δ*ald*_tac_ald strains growing on different NaF concentrations. (a) Growth curves of *P. putida* Δ*crcB*, Δ*crcB*Δ*3125*Δ*ald,* and Δ*crcB*Δ*3125*Δ*ald*_tac_ald strains growing on different NaF concentrations. 0.01 and 0.5 mM IPTG was used to induce the *tac*-promoter. Averages of three different biological replicas with three different technical parallels are presented with standard deviation. The background colours show the length of the lag-phase; the darker the background, the longer the lag-phase. (b) Maximum growth rate (μ) and *lag*-phase of *P. putida* Δ*crcB*, Δ*crcB*Δ*3125*Δ*ald,* and Δ*crcB*Δ*3125*Δ*ald*_tac_ald strains growing on different NaF concentrations with standard derivations.

(a)

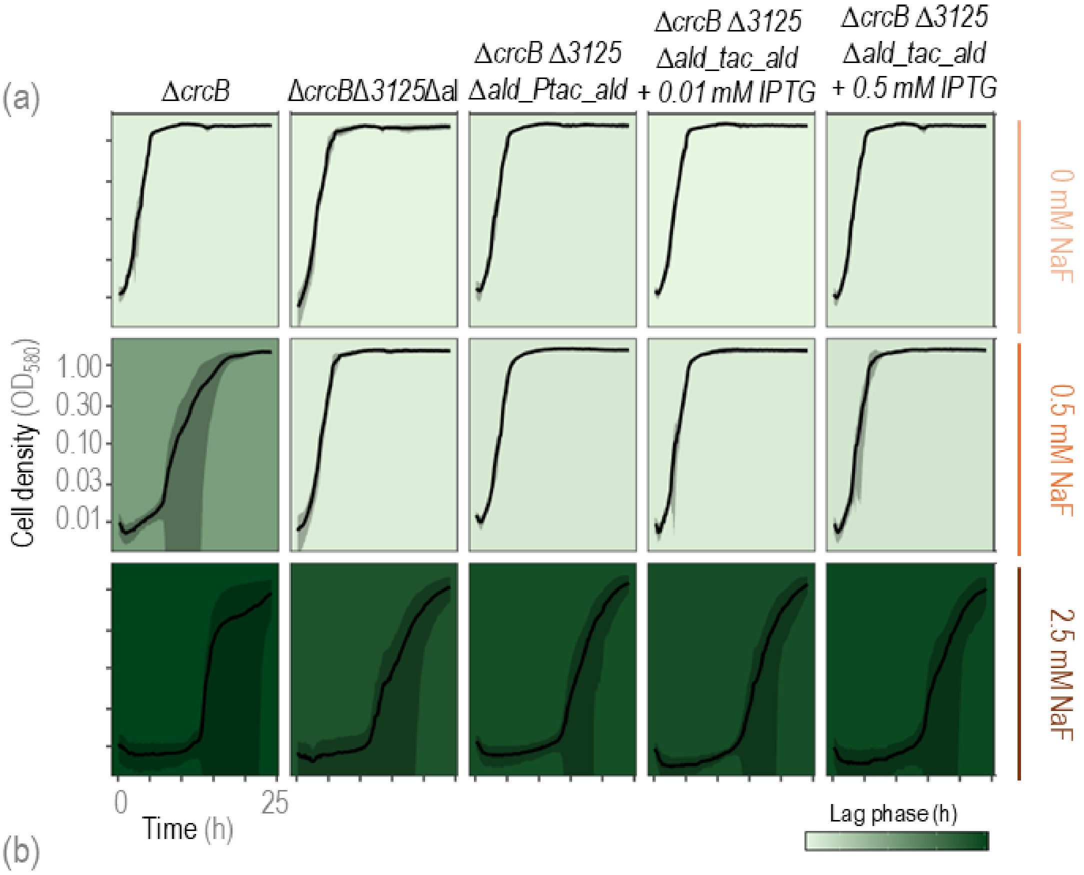

(b)

Parameter	NaF concn. (mM)	*P. putida* strain				
		Δ*crcB*	Δ*crcB*Δ*3125* Δald	Δ*crcB* Δ*3125*Δ*ald*_*tac*_*ald*		
				– IPTG	+ 0.01 mM IPTG	+ 0.5 mM IPTG
μ (h⁻¹)	0	1.5 ± 0.2	1.4 ±0.2	1.4 ± 0.2	1.5 ± 0.3	1.5 ± 0.2
	0.5	1.0 ± 0.2	1.4 ± 0.1	1.5 ± 0.2	1.6 ± 0.2	1.6 ± 0.2
	2.5	1.4 ± 0.4	1.2 ± 0.2	1.3 ± 0.1	1.3 ± 0.1	1.2 ± 0.2
Lag phase (h)	0	1.6 ± 0.4	1.2 ± 0.2	1.6 ± 0.3	1.5 ± 0.3	1.5 ± 0.2
	0.5	8.1 ± 1.8	1.9 ± 0.1	2.1 ± 0.3	2.0 ± 0.4	2.6 ± 0.7
	2.5	16.9 ± 3.6	14.8 ± 2.6	15.8 ± 2.0	15.8 ± 2.1	15.9 ± 2.2
